# Antibody acquisition models: A new tool for serological surveillance of malaria transmission intensity

**DOI:** 10.1038/srep19472

**Published:** 2016-02-05

**Authors:** Victor Yman, Michael T. White, Josea Rono, Bruno Arcà, Faith H. Osier, Marita Troye-Blomberg, Stéphanie Boström, Raffaele Ronca, Ingegerd Rooth, Anna Färnert

**Affiliations:** 1 Unit of Infectious Diseases, Department of Medicine Solna, Karolinska Institutet, Stockholm, Sweden; 2 MRC Centre for Outbreak Analysis & Modelling, Department of Infectious Disease Epidemiology, Imperial College, London, United Kingdom; 3 KEMRI-Wellcome Trust Research Programme, Centre for Geographical Medicine Research-Coast, Kilifi, Kenya; 4 Department of Public Health and Infectious Diseases, Parasitology Section, Sapienza University of Rome, Italy; 5 Department of Molecular Biosciences, the Wenner-Gren Institute, Stockholm University, Sweden; 6 Department of Biology, Federico II University, Naples, Italy; 7 Nyamisati Malaria Research Unit, Rufiji, Tanzania

## Abstract

Serology has become an increasingly important tool for the surveillance of a wide range of infectious diseases. It has been particularly useful to monitor malaria transmission in elimination settings where existing metrics such as parasite prevalence and incidence of clinical cases are less sensitive. Seroconversion rates, based on antibody prevalence to *Plasmodium falciparum* asexual blood-stage antigens, provide estimates of transmission intensity that correlate with entomological inoculation rates but lack precision in settings where seroprevalence is still high. Here we present a new and widely applicable method, based on cross-sectional data on individual antibody levels. We evaluate its use as a sero-surveillance tool in a Tanzanian setting with declining malaria prevalence. We find that the newly developed mathematical models produce more precise estimates of transmission patterns, are robust in high transmission settings and when sample sizes are small, and provide a powerful tool for serological evaluation of malaria transmission intensity.

In order to reduce the global malaria burden and achieve control, or even elimination, robust estimates of malaria transmission intensity are required for the strategic planning, implementation and evaluation of interventions^1^,^2^,^3^,^4^. Efficient monitoring of malaria transmission intensity depends on tools that produce reliable estimates across a wide range of transmission settings^5^,^6^. Such tools should preferably integrate information about both parasite and vector populations to capture the current level of transmission intensity as well as the transmission potential in areas where parasite carriage has decreased but vector populations persist^6^,^7^,^8^,^9^.

Traditionally, transmission intensity has been estimated by a variety of techniques such as spleen rates, parasite prevalence or entomological inoculation rates (EIR)^2^. EIR has been considered the gold standard among metrics^10^, but is expensive and labour intensive to evaluate and estimates are often imprecise (especially when transmission is low) due to marked heterogeneity of both malaria transmission and vector distribution^10^,^11^,^12^. In addition, single-time point evaluation of parasite prevalence or EIR provides limited information about past transmission intensity^13^,^14^. Cumulative exposure to *P. falciparum*, however, can be estimated by evaluation of antibody responses to *P. falciparum* blood-stage antigens^15^,^16^,^17^. In addition, exposure to *Anopheles* mosquitoes can be evaluated through antibody responses to *An. gambiae* salivary gland protein 6 (gSG6)^18^,^19^,^20^.

Existing methods for serological evaluation of malaria transmission have largely been based on cross-sectional data on antibody prevalence and on estimation of seroconversion rates (SCR) using serocatalytic models as shown by Drakeley *et al.*^15^. Although SCR based estimates have been extended to evaluate temporal changes^21^,^22^,^23^ and provide robust information about medium and long-term trends of transmission intensity, they are insensitive to changes in high transmission settings and to smaller and more short-term trends due to the quick acquisition of antibody responses and the long half-life of seropositivity to many blood-stage antigens^15^,^24^,^25^. The sensitivity and precision can be improved by selecting antigens that are less immunogenic or to which seropositivity is more short-lived^15^,^26^ and, as duration of seropositivity appears to be age-dependent^27^, by focusing analysis on antibody responses among children only and by using data from multiple cross-sectional surveys^28^. Nonetheless, recent changes in exposure, and changes occurring in high exposure settings, are better reflected by population antibody levels^29^,^30^,^31^. SCR based estimates could potentially be further improved by the application of a model that makes use of the data on individual antibody levels by assuming that antibody levels increase as a function of age, and that the rate at which they are boosted by exposure can be used as a marker of transmission intensity.

Here we aim to improve serological estimates of malaria transmission intensity by developing new models based on cross-sectional data on individual antibody levels. We evaluate transmission changes in a rural Tanzanian village that has experienced a gradual decline of *P. falciparum* prevalence from 1985 to 2010, but where lack of surveillance between 2000 and 2009 hinders efforts to fully elucidate patterns of malaria transmission intensity^32^ (Fig. 1). To maximise the sensitivity to detect transmission changes, we included children 1–16 years old participating in cross-sectional surveys conducted in 1999 (n = 313) and 2010 (n = 355) and measured antibody responses to recombinant *P. falciparum* antigens (MSP-1, MSP-2, MSP-3, AMA-1) and gSG6. *P. falciparum* antigens were selected based on previous evaluation with serocatalytic models^15^,^20^,^27^. We compare the performance of the newly developed models, referred to as antibody acquisition models, to previously validated serocatalytic models. We show that the antibody acquisition models increase precision in transmission estimates and provide a powerful and widely applicable new tool for serological surveillance of malaria transmission dynamics.

## Results

The prevalence of *P. falciparum* infection in children age 1–16 was 77.1% (95% CI: 71.2, 81.8) in 1999 and 23.7% (95% CI: 15.7, 24.3) in 2010 when evaluated by real-time PCR^32^. We defined the threshold for seropositivity as the mean reactivity, plus three standard deviations, of sera from unexposed Swedish donors (see Methods). In addition, we evaluated an alternative threshold for seropositivity defined using finite mixture models (see Methods). Results from serocatalytic models fitted to seroprevalence data based on the alternative threshold are presented in full as supplementary information. For all antigens, both prevalence (χ^2^-test: all *P* < 0.001) and levels (Mann-Whitney-U-test: all *P *<* *0.001) of antibodies were higher in 1999 than 2010. Seroprevalence and levels of antibodies to *P. falciparum* antigens increased with age at both surveys, except for MSP-1_19_, MSP-3_3D7 and MSP-3_k1 in the 2010 cross-section where levels were similar across age groups. Seroprevalence and antibody levels to gSG6 did not show discernible age trends at either survey. Data on antibody levels were approximately log-normally distributed for all antigens (see Supplementary Fig. S3 online) and highly correlated between the two allelic variants of AMA-1 and MSP-3, while less correlated for MSP-2 (see Supplementary Fig. S4 online).

### Modelling serological data

**Figure 1:**
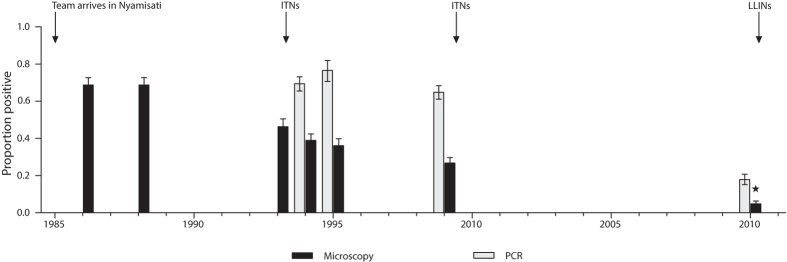
All age parasite prevalence in Nyamisati 1985–2010 by microscopy and species specific real-time PCR^32^. (*) Microscopy prevalence in 2010 was estimated from PCR data using the prevalence estimation tool developed by Okell *et al.* Nat Commun. 2012. Arrows indicate the time-points for distribution of insecticide treated nets (ITNs) and long-lasting insecticidal nets (LLINs).

Based on our previous knowledge of the gradual reduction in parasite prevalence in Nyamisati^32^ (Fig. 1), we considered three functional forms for the changing pattern of malaria transmission over time: (i) constant transmission; (ii) a sharp stepwise reduction; (iii) a linear decline. The serocatalytic models and the new antibody acquisition models were fitted to the data for each antigen and transmission profile using maximum-likelihood estimation (see Methods). For each individual antigen and transmission pattern, models were fitted simultaneously to data from the two cross-sectional surveys.

#### Serocatalytic models

**Figure 2:**
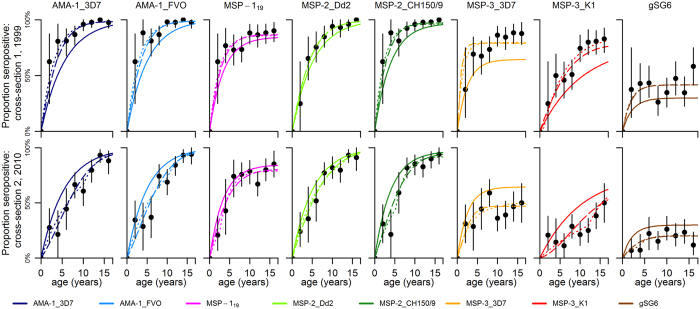
Best-fit serocatalytic models. Black points denote the proportion of seropositive individuals and vertical bars denote 95% confidence intervals. Model 1: stable transmission (solid lines). Model 2: stepwise reduction in transmission (dotted lines). Model 3: linear reduction in transmission (dashed lines).

**Figure 3:**
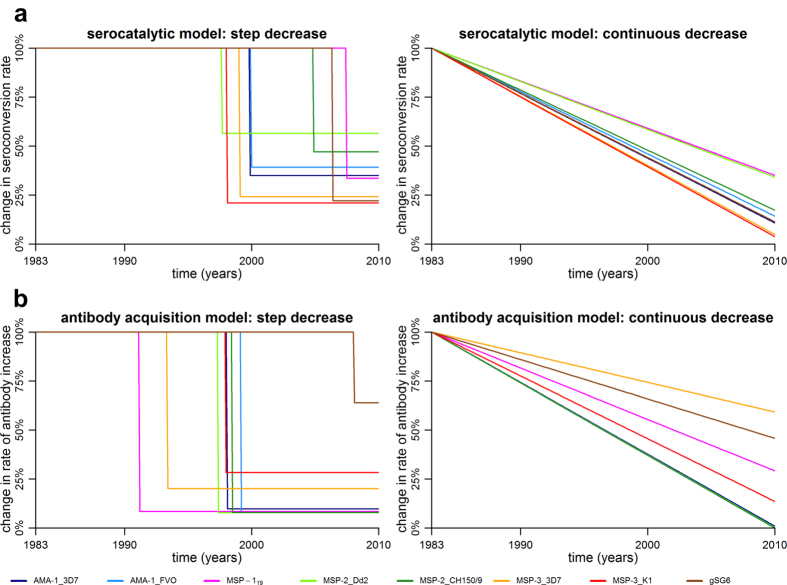
(**a**) Estimated changes in historical transmission from serocatalytic models (model 2 and model 3). (**b)** Estimated changes in historical transmission from antibody acquisition models (model 2 and model 3).

In Fig. 2 we present age-seroprevalence plots and the three models fitted for each of the antigens at both cross-sections. Estimates of SCR (*λ*) and seroreversion rates (*ρ*) for each model and antigen are presented in Table 1 together with the estimated time (*t*_*c*_) and magnitude of reduction (*γ*). As expected, model 1 provided poor fit to data for all antigens and a visual assessment of the fitted models in the age-seroprevalence plots indicated that model 1 underestimated the proportion of seropositives in 1999 while simultaneously overestimating the proportion of seropositives in 2010 (Fig. 2). The stepwise decrease model (model 2) estimated an approximate 44–79% reduction of SCR for *P. falciparum* antigens from 1983–2010 (Table 1). The reduction was estimated to have occurred between 1997 and 2007, with most point estimates clustered between 1997 and 2000 (Table 1, Fig. 3a). AMA-1_3D7, AMA-1_FVO and MSP-3_K1 provided precise estimates of “time of change” (*t*_c_) whereas estimates from MSP-1_19_, MSP-2_Dd2, MSP-2_CH150/9 and MSP-3_3D7 and gSG6 showed substantial uncertainty (Table 1). Model 3 estimated high initial SCRs in 1983 and large reductions for all antigens between 1983 and 2010. The estimated reduction ranged from 65–96% for the *P. falciparum* antigens (Table 1, Fig. 3a). Model comparison using the Akaike Information Criterion (AIC) revealed that models 2 and 3, which capture reductions in transmission, provide considerably better fits to the data than model 1 (Table 1). According to the classification suggested by Burnham and Anderson^33^ (∆AIC < 2: substantial support for alternative models; ∆AIC = 4–7: considerably less support; ∆AIC > 10: no support), there was sufficient information only to determine that model 2 was the superior model for AMA-1 and MSP-3_k1 whereas for the other antigens, AIC-values were similar between models 2 and 3 (Table 1). Serocatalytic models fitted to seroprevalence data based on the alternative threshold for seropositivity estimated lower SCR and higher seroreversion rates, but results were in all other aspects similar and the alternative choice of threshold did not affect model performance (see Supplementary Fig. S5 and Supplementary Table S1 online).

#### Antibody acquisition models

**Figure 4:**
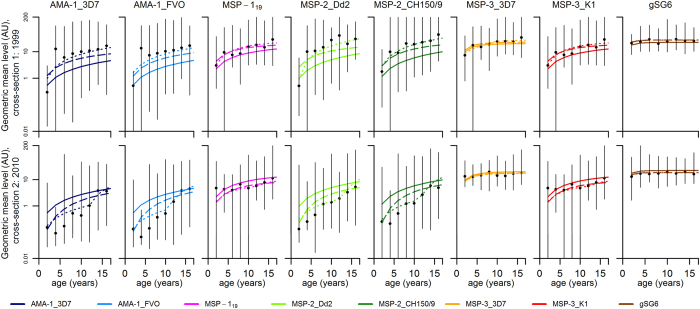
Best-fit antibody acquisition models. Black points denote geometric mean antibody levels in arbitrary units (AU) and vertical bars denote the 95% range of the data. Model 1: stable transmission (solid lines). Model 2: stepwise reduction in transmission (dotted lines). Model 3: linear reduction in transmission (dashed lines).

Antibody acquisition models were designed to capture the rate of increase in antibody levels with age and provide an alternative measure of transmission intensity. Age group-specific geometric mean antibody levels and the antibody acquisition models fitted to data for each of the antigens are presented in Fig. 4. Maximum likelihood parameter estimates of antibody acquisition (a) and decay rates (*r*), timing (*t*_*c*_) and magnitude of reduction (*γ*) are shown in Table 2. Model 2 (i.e. stepwise decrease model) provided superior fit to data (with the lowest AIC values) compared to both model 3 and model 1 for all *P. falciparum* antigens (Table 2). Model 2 estimated reductions of antibody acquisition rates for *P. falciparum* antigens ranging from 72–92% between 1983 and 2010 (Table 2, Fig. 3b) with a stepwise reduction estimated to have occurred between 1997 and 1999 for antigens AMA-1_FVO, AMA-1_3D7, MSP-2_Dd2, MSP-2_CH150/9 and MSP-3_K1 (Table 2, Fig. 3b). MSP-1_19_ and MSP-3_3D7, showing no age-trend in antibody levels at the second cross-section, as well as gSG6, with no age-trend at either cross-section, each provided estimates with considerable uncertainty (Table 2, Fig. 3b). Model 3 estimated reductions of antibody acquisition rates from 41–100%, approaching 100% for several of the *P. falciparum* antigens (AMA-1_3D7, AMA-1_FVO, MSP-1_19_, MSP-2_Dd2, and MSP-2_CH150/9), indicating zero transmission in 2010 and thus a poor choice of pattern of transmission intensity (Table 2, Fig. 3b).

### Model comparison: Antibody acquisition models versus serocatalytic models

The serocatalytic models use continuous antibody data that has been dichotomised while the antibody acquisition models use data on the actual individual antibody level and age. The different types of data restrict a formal statistical comparison; however, the performance of the models can be evaluated and compared using profile likelihood plots (see Supplementary Information online). The antibody acquisition models generally provided a better and more consistent fit to data than the serocatalytic models. The difference is evident from the profile likelihood plots, in particular for the parameter “time of change” (*t*_*c*_) for serocatalytic and antibody acquisition model 2 (see Supplementary Fig. S1 and S2 online). Although the point estimates of parameter *t*_*c*_ were overall similar for both serocatalytic and antibody acquisition models, the larger amount of data utilised by the antibody acquisition models gave better fit and provided higher precision of the estimates as indicated by the much more narrow confidence intervals (Table 1 and 2).

We performed a complementary sensitivity analysis (see Supplementary Figs S6 and S7 online), using both simulated data and the data for AMA-1_FVO, to evaluate the robustness of serocatalytic and antibody acquisition model 2 to reductions in the sample size. Reductions in sample size (e.g. to 100 or 50 samples per cross-section) reduced both accuracy and precision of serocatalytic model estimates of parameter *t*_*c*_ whereas it only slightly affected the precision of the antibody acquisition model, illustrating the robustness of the antibody acquisition model.

## Discussion

From 1985 to 2010, the population of Nyamisati village experienced a gradual decline of *P. falciparum* prevalence, but lack of surveillance between 1999 and 2010 hinders efforts to elucidate patterns of malaria transmission intensity and thereby to identify factors that have contributed to the decline^32^. Through mathematical modelling of serological data, we evaluate historical transmission patterns and confirm a reduction in *P. falciparum* transmission intensity. Serocatalytic models indicated a decrease of malaria transmission intensity but could not determine if the reduction pattern was more likely to be stepwise or linear. The new antibody acquisition models, using the complete data on antibody levels, increase both power and precision and enabled us to establish that a stepwise reduction in transmission decrease was most likely.

The two modelling approaches have strengths and weaknesses, which are outlined in Table 3. In high and moderate transmission settings, serocatalytic models are often limited by the long half-life of seropositivity and a plateau of very high seroprevalence reached already among young children^6^. This is likely to have contributed to the uncertainty of serocatalytic model parameter estimates in the present study. However, population antibody levels do not saturate as seroprevalence does, and if transmission is reduced antibody levels will drop while seropositivity remains high^29^,^31^,^34^,^35^. The antibody acquisition models incorporate insights from longitudinal antibody dynamics^31^ and limit the loss of information that occurs when continuous antibody data is dichotomised^36^, thereby providing additional power to detect changes in transmission intensity. The antibody acquisition models performed better than the corresponding serocatalytic models in this context of high to moderate transmission intensity. Both serocatalytic and antibody acquisition model 2 provided point-estimates of “time of change” (*t*_*c*_) that, for the majority of the antigens, clustered around 1997 to 2000. However, the antibody acquisition model provided more precise estimates, due to a higher power to detect changes when seroprevalence is high^22^,^29^.

Exact determination of transmission dynamics requires frequent and longitudinal sampling. However, here, as well as in many other settings, we are limited to cross-sectional data from single or only a few time-points. The presented models are plausible simplifications of the dynamics of transmission intensity in Nyamisati; nonetheless, they provide robust estimates of transmission intensity patterns. The 72–92% stepwise reduction of malaria transmission intensity, estimated by both serocatalytic and antibody acquisition models to have occurred around 1998 to 1999, coincided with the distribution of ITNs after the cross-sectional survey in 1999. The actual ITN coverage after the intervention is not known, however, the distribution of 900 bed nets in a population of approximately 1500 individuals would have provided more or less universal coverage (assuming one net protects 1.8 individuals^37^). Although parasite prevalence in Nyamisati decreased gradually during the late 1980’s and early1990’s^32^, and although factors other than the introduction of short-lasting ITNs could have contributed to the low level of *P. falciparum* and *Anopheles* exposure observed in Nyamisati in 2010^9^, our results strongly support that the single largest change in transmission intensity is related to the distribution of ITNs in 1999.

The lack of EIR data from the Nyamisati area is a limitation of the study. We therefore used gSG6 antibody responses as a proxy for vector exposure^19^,^20^. Antibody responses to gSG6 did not show any significant increase with age but the the age-dependent tolerance that has been shown elsewhere was not observed^38^. The lack of an age trend may be related to the short-lived nature of the response^39^ and limits their value in analysis using the present modelling approach. Nonetheless, we observe a significant reduction of both overall prevalence and levels of antibodies to gSG6 from 1999 to 2010 suggesting a substantial reduction of *Anopheles* exposure in Nyamisati.

The inclusion of antigens that have been previously evaluated in serocatalytic models^15^,^23^,^27^ allows us to compare the value of these antigens when using antibody acquisition models. MSP-1_19_ has been proposed suitable for a wide range of transmission intensities^21^, while the more immunogenic AMA-1 suggested for low transmission settings^21^,^27^. Here, antibody responses to MSP-1_19_ and MSP-3 antigens were not particularly informative. The lack of age trend in antibody data for these antigens in this setting, particularly at the second cross-section is a major limitation for their use in both the serocatalytic and the antibody acquisition models and affects both accuracy and precision of model parameter estimates. For the MSP-2 proteins, antibody acquisition models, but not serocatalytic models, provided parameter estimates with a low level of uncertainty. A relatively low correlation of MSP-2_Dd2 and MSP-2_CH150/9 responses was observed and the highly polymorphic nature of the protein may pose a problem in some settings^40^. AMA-1 provided the most precise parameter estimates and the most consistent results between both the serocatalytic and antibody acquisition models. There was little saturation of AMA-1 antibody levels with increasing age and a high correlation between responses to the two allelic variants. These findings indicate the suitability of AMA-1 for a wide range of transmission settings when applying both serocatalytic and antibody acquisition models.

Antibody prevalence data has been used successfully to model the force of infection of malaria in different settings^15^,^21^,^22^,^23^ as well as several other infectious diseases, e.g. dengue^41^,^42^ and trachoma^43^. Estimates of malaria SCR based on longitudinal and cross-sectional data have been shown to provide consistent results^44^. Here we describe how cross-sectional data on antibody levels can be used to model malaria transmission intensity by assuming that the rate at which antibody levels are acquired and decay can be used as a marker for transmission intensity. The method was designed for malaria but can be applied to other pathogens. We show that the use of antibody acquisition models, which limits the loss of information and increases statistical power, enables sensitive detection of transmission changes and improves precision in estimates of malaria transmission intensity trends. We conclude that the new antibody acquisition models provide superior precision compared to serocatalytic models in settings of moderate and intense transmission where seroprevalence is high. We propose that the antibody acquisition models provide a new powerful tool for serological surveillance of short-, medium- and long-term trends of malaria transmission and highlight their potential for infectious disease surveillance.

## Material and Methods

### Study site and cohort

The study site, cohort and malaria prevalence in the area have been recently described in detail^32^. Briefly, the study was conducted in Nyamisati, a rural fishing village situated in the Rufiji river delta, Tanzania, where malaria transmission is perennial with some increase following the two rainy seasons. The predominant malaria vectors in the area were species belonging to the *An. gambiae* complex as well as *An*. *funestus*^45^. A research team monitored malaria transmission and maintained a primary health care unit from 1985 until 2000. All malaria cases were continuously recorded through a passive case detection system 1986–88 and 1993–99. Cross-sectional surveys were performed yearly from 1985 to 1988, from 1993 to 1999 and in 2010. Surveys were conducted at the start of the major rainy season and included clinical examination and collection of venous blood samples. There was no malariometric surveillance between 2000 and 2009. Insecticide treated nets (ITNs) were distributed after the surveys in 1993 (300 ITNs to pregnant women and young children) and in 1999 (900 ITNs). The estimated access to bed nets after the surveys, assuming average protection of 1.8 individuals per net^37^, was 3% in 1985, 45% in 1993–1994 and 100% in 1999. Additionally, long-lasting insecticidal nets were distributed after the survey in 2010. Indoor residual spraying has not been used in the area. EIR data has not been collected at the study site. Prevalence of *P. falciparum* declined gradually from 1985 to 2010 (Fig. 1). Prevalence by microscopy decreased from 1985 to 1999, with only minor changes in real-time PCR prevalence. A large reduction in parasite prevalence by PCR was observed from 1999 to 2010^32^. To maximise the sensitivity to detect transmission changes, children (1–16 years of age) participating in two cross-sectional surveys conducted in 1999 (n = 313) and 2010 (n = 355), were included in the present study^27^,^28^. Children below 1 years of age were excluded due to the potential presence of maternal antibodies.

Ethical approval was obtained from the Nyamisati village board, the Ethical Review board of the National Institute for Medical Research in Tanzania and by the Regional Ethical Committee at Karolinska Institutet, Sweden (Dnr. 00–084 and 2012/1151–32). Informed consent was obtained from each participant and/or their guardians and experiments were carried out in accordance with approved guidelines.

### Antibody assays

Detection and quantitation of IgG antibodies against *P. falciparum* antigens and gSG6 were performed using a multiplex bead based immunoassay^46^ and ELISA^19^,^47^, respectively. The recombinant *P. falciparum* antigens included the 19 kDa fragment of merozoite surface protein 1 (MSP-1_19_)^48^, two allelic forms of each of merozoite surface protein 2 (MSP-2) (CH150/9 and Dd2)^49^, merozoite surface protein 3 (MSP-3) (K1 and 3D7)^50^ and apical membrane antigen 1 (AMA-1) (3D7 and FVO)^51^. The threshold for seropositivity was defined as the mean fluorescent intensity (MFI), or mean optical density (OD), plus three standard deviations of a negative control of pooled plasma from malaria unexposed Swedish donors. MFIs and ODs were converted to a relative concentration in arbitrary units (AU) using IgG standards run on each plate (see Supplementary Information online).

An alternative threshold for seropositivity was defined by fitting finite mixture models^52^,^53^ to the log-transformed antibody data distributions assuming a two-component Gaussian mixture according to a previously described method (see Supplementary Information online)^23^,^54^,^55^.

### Antibody models

#### Serocatalytic models

Cross-sectional data on age-specific prevalence and levels of antibodies from children 1–16 years old are assumed to represent cumulative exposure to the antigen during the child’s lifespan, thereby allowing us to estimate transmission patterns during the 16-year period prior to each cross-section. Serological data on children aged 1–16 from two cross-sections in 1999 and 2010, provide information on transmission from 1983 to 1999 for cross-section 1, and from 1994 to 2010 for cross-section 2. Denote *t* to be the time after the start of the transmission period (in 1983), and *t*_end_ the time at the end of the period (in 2010). We use the seroconversion rate *λ* as a marker for transmission intensity^15^,^21^. We consider three profiles for changes in transmission intensity over this time period:

- Model 1: Constant transmission
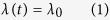
- Model 2: Sharp drop in transmission at time t_c_
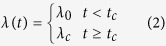
- Model 3: Linear reduction in transmission
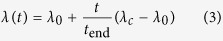

If seronegative individuals become seropositive at rate *λ*(*t*), and if seropositive individuals revert at rate *ρ*, then the proportion of seropositive individuals in a cohort *P* is described by the differential equation


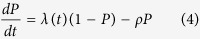


This equation can be solved for the three transmission profiles to give estimates of the proportion of individuals of age *a* seropositive at each cross-section.

- Model 1: Constant transmission
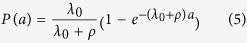
- Model 2: Sharp drop in transmission at time t_c_
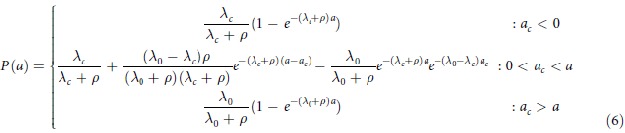
where *a*_*c*_ is the age of the child at the time of the drop in transmission (*a*_*c*_ = *a* + *t*_*c*_ − 16 for children from cross-section 1, and *a*_*c*_ = *a* + *t*_*c*_ − 27 for children from cross-section 2)
- There is no analytical solution for Model 3 so it must be solved numerically

The magnitude of the reduction in seroconversion rate is defined as *γ (*=*λ*_*c*_/*λ*_*0*_).

The serocatalytic model was fitted to age-dependent data on sero-positivity status using a binomial likelihood function. The log-likelihood was maximised to obtain the maximum likelihood parameter estimate (see Supplementary Information online). 95% confidence intervals were calculated using the likelihood ratio test. Profile likelihood plots are presented in Supplementary Figure S1 online.

#### Antibody acquisition models

In the serocatalytic models described above all data on the continuous antibody levels has been reduced to a dichotomous positive or negative value. Here, we describe how the full data on antibody levels can be utilised. We assume that the rate at which antibody levels are acquired α can be used as a marker for transmission intensity^31^. If an individual’s antibody level is boosted at rate *α*(*t*) and decays at rate *r* then antibody levels can be described by the following differential equation


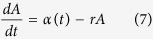


We assume α(*t*) corresponds to the three transmission profiles outlined above.

- Model 1: Constant transmission
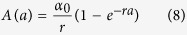
- Model 2: Sharp drop in transmission at time t_c_
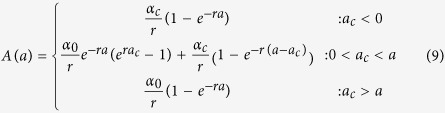
where *a*_*c*_ is the age of the child at the time of the drop in transmission.
- Model 3: Linear reduction in transmission
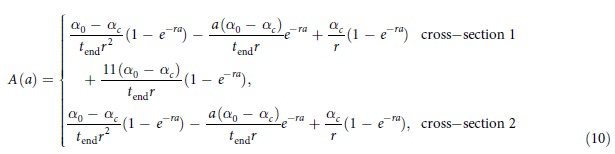

The magnitude of reduction in antibody acquisition rates is defined as *γ* ( = *α*_*c*_/*α*_*0*_).

The antibody acquisition model was fitted to age-dependent antibody level data from both cross-sections by assuming the geometric mean antibody level at age *a* is *A(a)* and that antibody levels are log-normally distributed in the cohort with standard deviation on the log scale *σ*. The log-likelihood was maximised to obtain the maximum likelihood parameter estimate (see Supplementary Information online). 95% confidence intervals were calculated using the likelihood ratio test. Profile likelihood plots are presented in Supplementary Figure S2 online.

## Additional Information

**How to cite this article**: Yman, V. *et al.* Antibody acquisition models: A new tool for serological surveillance of malaria transmission intensity. *Sci. Rep.* **6**, 19472; doi: 10.1038/srep19472 (2016).

## Supplementary Material

Supplementary Information

## Figures and Tables

**Table 1:** Serocatalytic model parameter estimates.

Antigen	Model	*λ*_*0*_	*γ*	*ρ*	*t*_*c*_	Log- likelihood	AIC
AMA-1_3D7	M1	0.17 (0.16, 0.22)	–	0.0 (0.0, 0.02)	–	−65.74	135.48
	M2	0.32 (0.26, 0.44)	0.35 (0.26, 0.46)	0.003 (0.0, 0.015)	1999 (1997, 2001)	−31.59	**71.19**
	M3	0.51 (0.39, 0.69)	0.11 (0.04, 0.21)	0.004 (0.0, 0.016)	–	−34.92	75.85
AMA-1_FVO	M1	0.20 (0.18, 0.24)	–	0.0 (0.0, 0.01)	–	−60.30	124.60
	M2	0.34 (0.29, 0.43)	0.39 (0.29, 0.52)	0.003 (0.0, 0.013)	1999 (1997, 2001)	−33.48	**74.96**
	M3	0.53 (0.41, 0.74)	0.14 (0.06, 0.27)	0.004 (0.0, 0.015)	–	−36.11	78.23
MSP-1_19_	M1	0.25 (0.19, 0.34)	–	0.045 (0.02, 0.08)	–	−42.20	88.41
	M2	0.36 (0.26, 0.56)	0.33 (0.26, 0.73)	0.06 (0.03, 0.10)	2007 (1997, 2009)	−35.53	**79.07**
	M3	0.50 (0.31, 0.91)	0.35 (0.17, 0.66)	0.05 (0.03, 0.09)	–	−36.88	79.77
MSP-2_Dd2	M1	0.19 (0.17, 0.22)	–	0.0 (0.0, 0.01)	–	−38.87	81.75
	M2	0.26 (0.21, 0.41)	0.56 (0.31, 0.75)	0.0 (0.0, 0.01)	1997 (1994, 2006)	−29.60	67.20
	M3	0.35 (0.26, 0.51)	0.34 (0.18, 0.57)	0.002 (0.0, 0.016)		−29.81	**65.62**
MSP-2_CH150/9	M1	0.24 (0.21, 0.31)	–	0.007 (0.0, 0.02)	–	−48.32	100.65
	M2	0.43 (0.29, 0.64)	0.28 (0.14, 0.60)	0.016 (0.0, 0.03)	2004 (1997, 2006)	−31.52	71.04
	M3	0.64 (0.44, 1.06)	0.17 (0.07, 0.34)	0.011 (0.0, 0.027)	–	−31.74	**69.49**
MSP-3_3D7	M1	0.26 (0.18, 0.45)	–	0.14 (0.08, 0.28)	–	−85.19	174.39
	M2	0.62 (0.31, 2.69)	0.24 (0.04, 0.34)	0.16 (0.06, 0.61)	1999 (1996, 2008)	−43.31	94.62
	M3	2.15 (1.06, 8.44)	0.05 (0.01, 0.11)	0.26 (0.15, 0.70)	–	−44.24	**94.48**
MSP-3_K1	M1	0.07 (0.06, 0.12)	–	0.02 (0.0, 0.09)	–	−94.18	192.37
	M2	0.16 (0.11, 0.26)	0.21 (0.14, 0.29)	0.03 (0.0, 0.06)	1998 (1996, 1999)	−51.26	**86.50**
	M3	0.25 (0.16, 0.41)	0.04 (0.0, 0.11)	0.04 (0.01, 0.09)	–	−48.16	102.32
gSG6	M1	0.14 (0.06, 4.18)	–	0.32 (0.12, 10.0)	–	−56.82	117.64
	M2	0.24 (0.11, 3.52)	0.22 (0.01, 0.46)	0.34 (0.15, 1.68)	2006 (1996, 2009)	−37.68	83.36
	M3	0.47 (0.21, 4.33)	0.11 (0.04, 0.23)	0.35 (0.17, 4.10)	–	−38.03	**82.07**

Maximum likelihood parameter estimates and 95% confidence intervals for serocatalytic models fitted to cross-sectional age-specific seropositivity data. *λ*_*0*_ is the seroconversion rate, *γ (*=*λ*_*c*_*/λ*_*0*_) is the reduction in transmission, *ρ* is the seroreversion rate, *t*_*c*_ is the estimated time-point (calendar-year) of drop in transmission, *log-likelihood* is the maximised log-likelihood of the model and *AIC* is the Akaike Information Criterion value. A bold font indicates the smallest AIC for each of the antigens. Confidence Intervals were defined using profile-likelihood methods.

**Table 2:** Antibody acquisition model parameter estimates.

Antigen	Model	*α*_*0*_	*γ*	*r*	*t*_*c*_	*σ*	Log-likelihood	AIC
AMA-1_3D7	M1	0.27 (0.24, 0.34)	–	0.0 (0.0, 0.03)	–	2.18 (2.06, 2.29)	−2026.17	4058.35
	M2	0.89 (0.66, 1.32)	0.09 (0.07, 0.14)	0.0 (0.0, 0.05)	1998 (1997, 1999)	1.92 (1.82, 2.02)	−1942.73	**3895.45**
	M3	1.55 (0.91, 2.72)	0.009(0.001,0.04)	0.10 (0.01, 0.20)	–	1.97 (1.88, 2.08)	−1961.93	3931.85
AMA-1_FVO	M1	0.28 (0.24, 0.34)	–	0.0 (0.0, 0.02)	–	2.23 (2.11, 2.35)	−2052.93	4111.85
	M2	0.95 (0.66, 1.47)	0.08 (0.06, 0.12)	0.0 (0.0, 0.06)	1998 (1997, 2000)	1.93 (1.81, 2.06)	−1957.98	3925.96
	M3	1.48 (0.72, 2.53)	0.003(0.001,0.03)	0.08 (0.0, 0.18)	–	2.00 (1.91, 2.11)	−1980.09	3968.18
MSP-1_19_	M1	0.47 (0.34, 0.71)	–	0.07 (0.0, 0.18)	–	1.67 (1.59, 1.77)	−1998.72	4003.44
	M2	7.8. (1.2, 45.5)	0.084 (0.016, 0.28)	0.23 (0.02, 0.45)	1991 (1988, 2002)	1.64 (1.55, 1.72)	−1987.69	**3985.38**
	M3	1.19 (0.71, 2.01)	0.29 (0.17, 0.49)	0.13 (0.05, 0.25)	–	1.65 (1.57, 1.74)	−1988.70	3985.40
MSP-2_Dd2	M1	0.50 (0.44, 0.59)	–	0.0 (0.0, 0.02)	–	2.03 (1.92, 2.14)	−2381.28	4768.56
	M2	1.96 (1.56, 2.92)	0.08 (0.06, 0.11)	0.0 (0.0, 0.05)	1997 (1996, 1998)	1.70 (1.61, 1.81)	−2263.92	**4537.84**
	M3	3.37 (2.02, 5.58)	0.002 (0.001,0.2)	0.12 (0.04, 0.22)	–	1.79 (1.69, 1.88)	−2296.06	4600.12
MSP-2_CH150/9	M1	0.58 (0.49, 0.71)	–	0.0 (0.0, 0.02)	–	2.26 (2.14, 2.39)	−2562.06	5130.12
	M2	1.95 (1.52, 3.28)	0.08 (0.06, 0.11)	0.0 (0.0, 0.06)	1998 (1997, 1999)	1.95 (1.84, 2.06)	−2463.86	4937.71
	M3	3.64 (2.13, 6.37)	0.0 (0.0, 0.02)	0.11 (0.02, 0.21)	–	2.01 (1.91, 2.11)	−2483.66	4975.31
MSP-3_3D7	M1	6.80 (4.53, 12.97)	–	0.34 (0.19, 0.72)	–	1.17 (1.12, 1.24)	−2976.95	5959.90
	M2	29.98 (3.0,100)	0.08 (0.07,0.15)	0.35 (0.31,0.48)	1992 (1990, 1993)	1.15 (1.10,1.22)	−2966.78	5943.55
	M3	10.59 (6.18, 22.8)	0.59 (0.42, 0.86)	0.38 (0.22, 0.79)	–	1.16 (1.10, 1.24)	−2973.43	**5954.87**
MSP-3_K1	M1	1.20 (0.84, 1.89)	–	0.06 (0.0, 0.17)	–	1.81 (1.71, 1.90)	−2706.36	5418.72
	M2	2.90 (1.87, 6.69)	0.28 (0.18, 0.39)	0.10 (0.03, 0.22)	1998 (1996, 2001)	1.73 (1.63, 1.82)	−2675.38	5360.75
	M3	4.99 (3.07, 9.0)	0.14 (0.07, 0.23)	0.17 (0.09, 0.33)	–	1.74 (1.65, 1.84)	−2680.85	5369.70
gSG6	M1	18.2 (12.7, 32.1)	–	0.82 (0.55, 1.50)	–	0.69 (0.65, 0.73)	−2677.39	**5360.78**
	M2	27.6 (17.1, 39.4)	0.64 (0.42,0.75)	1.02 (0.60,1.50)	2008 (1997, 2009)	0.66 (0.63,0.71)	−2651.88	5313.75
	M3	37.25 (23.9, 42.5)	0.46 (0.38, 0.55)	0.96 (0.65, 1.19)	–	0.66 (0.63, 0.71)	−2652.21	**5312.41**

Maximum likelihood parameter estimates and 95% confidence intervals for antibody acquisition models fitted to cross-sectional antibody levels data. *α*_*0*_ is the rate of antibody boosting, *γ* (= *α*_*c*_/*α*_*0*_) is the reduction in transmission, *r* is the rate of antibody decay, *t*_*c*_ is the estimated time-point (calendar year) of drop in transmission, *σ* is the standard deviation of the antibody levels on the log scale, *log-likelihood* is the maximised log-likelihood of the model and *AIC* is the Akaike Information Criterion value. A bold font indicates the smallest AIC for each of the antigens. Confidence Intervals were defined using profile-likelihood methods.

**Table 3:** Comparative strengths and weaknesses of the serocatalytic and antibody acquisition models.

Serocatalytic models	Antibody acquisition models
**Strengths**	**Strengths**
• Effective at low transmission intensities when seropositivity is a good indicator of exposure.	• Uses all available data.
• Data from multiple antigens can be combined.	• Effective also at high transmission intensities where all individuals are seropositive.
**Weaknesses**	**Weaknesses**
• Data is compressed into a binary seronegative/seropositive state.	• Difficult to combine data from multiple antigens.
• Choice of seropositivity threshold is arbitrary.	• May not be applicable if data are not log-normally distributed, e.g. if there are many zero measurements.
