# Supplementary material for: Antibody acquisition models: A new tool for serological surveillance of malaria transmission intensity
Source: Sci Rep. 2016 Feb 5;6:19472. doi: 10.1038/srep19472 (PMC4984902; doi:10.1038/srep19472)
Supplement: Supplementary Information [file srep19472-s1.pdf]

Supplementary Information

Antibody acquisition models: A new tool for serological surveillance of malaria transmission intensity

Victor Yman, Michael T White, Josea Rono, Bruno Arcà, Faith H Osier, Marita Troye-Blomberg, Stéphanie Boström, Raffaele Ronca, Ingegerd Rooth, Anna Färnert

Supplementary methods

Antibody assays

Detection and quantitation of IgG antibody responses by multiplex bead-based assay

A multiplex bead based immunoassay[1] was used to detect and quantify plasma IgG antibodies against seven recombinant *P. falciparum* asexual blood-stage antigens. Briefly, each of the recombinant antigens; merozoite surface protein (MSP-)1_{19}, two allelic forms of each of MSP-2 (CH150/9 and Dd2), MSP-3 (K1 and 3D7) and apical membrane antigen (AMA-)1 (3D7 and FVO); were coupled to a spectrally unique set of magnetic beads (Bio-Rad Laboratories, Hercules, CA, USA), using the BioPlex amine coupling kit (Bio-Rad Laboratories, Hercules, CA, USA) at an antigen concentration of 5 ng per 5000 beads. All antigen-coupled bead-sets were mixed and 5000 beads per antigen were distributed into each well of a Bio-Plex Pro Flat Bottom plate (Bio-Rad Laboratories, Hercules, CA, USA). One hundred µl of plasma, in a 1:1000 dilution in phosphate buffered saline (PBS) and Tween with 1% bovine serum albumin (BSA), was added to each well and incubated for 1 hour. After washing four times with PBS-Tween, 50µl per well of R-phycoerythrin-conjugated,

F(ab')2, goat anti-human IgG (Jackson ImmunoResearch Laboratories Inc., West Grove, PA, USA Inc.,) in 1:300 dilution in PBS-Tween with 1% BSA was added. After 30 minutes of incubation, beads were washed, resuspended in 50 µl of PBS-Tween with 1% BSA and analysed on the Bio-Plex200^{TM} instrument (Bio-Rad Laboratories, Hercules, CA, USA). A standard of serially diluted purified IgG from malaria immune donors was run on each plate.

Anopheles gambiae salivary gland protein 6 (gSG6) ELISA

ELISA for recombinant gSG6 was performed according to a previously described protocol[2] with some modifications. 96-well half-area microtiter plates (Costar® Corning, Tewksbury, MA, USA) were coated over night at 4 °C with 25 µl per well of recombinant gSG6 (5 µg/ml) in sodium-carbonate buffer. After washing four times with saline with 0.05% Tween 20, blocking was performed with 50 µl of sodium-carbonate buffer with 0.5% BSA for 2 hours at 37 °C. Plates were washed and 25 µl of plasma at 1:100 dilution in PBS-Tween with 0.5% BSA were added and incubated at 37 °C for 1 hour. After washing, IgG was detected using 25 µl/well of alkaline phosphatase conjugated goat-anti-human IgG (Mabtech, Nacka, Sweden), 1:2000 dilution, incubated for 30 minutes at 37 °C. After final washing the assay was developed with p-nitrophenyl phosphate disodium substrate (Sigma-Aldrich, St. Louus, MO, USA) and optical densities were read at 405 nm using a Vmax^{TM}Kinetic microplate reader (Molecular Devices, Sunnyvale, CA, USA). A standard curve was obtained through a sandwich ELISA of serially diluted highly purified human IgG (Jackson ImmunoResearch Laboratories Inc., West Grove, PA, USA) run on each plate[2].

Seropositivity threshold and antibody quantitation

All plasma samples were analysed in duplicate on each plate. Duplicates with a coefficient of variation (CV%) of above 20% were reanalysed. A negative and positive control, consisting of pooled plasma from adult malaria-unexposed Swedish donors and highly malaria exposed adult Kenyan donors from Kilifi district, respectively, were run in duplicates on each plate, each day of experiment. The mean fluorescent intensities (MFIs) and optical densities (ODs) were converted to a relative concentration in arbitrary units (AU) by interpolation from the standard curves using a five-parameter sigmoidal curve fitting. The threshold for seropositivity was defined as the mean MFI, or mean OD, of the negative control over all of the runs plus three standard deviations, a method that is straightforward, very widely used, sensitively identifies potentially seropositive individuals and in this case defines the Lower Limit of Detection (LLD) of the immunoassay. This method could be questioned for being potentially nonspecific due to the fact that unexposed individuals in endemic areas may have sera with a higher level of background reactivity compared to e.g. unexposed Europeans, possibly due to non-specific reactivity caused by previous exposure to other microorganisms[3,4]. We therefore evaluated an alternative threshold for seropositivity, generated by fitting finite mixture models (mixEM function from package mixtools in R version 3.2.2), assuming a two-component Gaussian mixture, to the log transformed antibody data distributions. As previously described, the threshold was then defined as the mean plus three standard deviations of the lower component[4]. Serocatalytic model fits and parameter estimates based on the alternative threshold for seropositivity are presented in detail in Supplementary Figure S5 and Supplementary Table S1. The two-component Gaussian mixture model approach, however, also has its limitations and may not perform well if there is not sufficient spread of the data, if the component distributions are not well separated, if sample size is small or if the distribution is a mixture of multiple components[5].

Model likelihoods

Serocatalytic model

The serocatalytic model was fitted to age-dependent data on sero-positivity status using a binomial likelihood. Assume that in age group i we have N_i samples at age a_i, k_i of which are seropositive. The likelihood that the model predicted seropositive proportion $P(a_i)$ fits the proportion seropositive observed from the data k_i/N_i is given by the binomial likelihood

$$L(\theta|N_i,k_i)=\binom{N_i}{k_i}P(a_i)^{k_i}(1-P(a_i))^{N_i-k_i}$$

where θ is the parameter vector. For model 1 we have $\theta=(\lambda_0,\rho)$, for model 2 $\theta=(\lambda_0,\lambda_c,t_c,\rho)$, and for model 3 $\theta=(\lambda_0,\lambda_c,\rho)$. The likelihood of the model fitting data on the proportion seropositive from all age groups in both cross-sections is given by

$$L(\theta|k^1_{1..I},N^1_{1..I},k^2_{1..I},N^2_{1..I})=\left(\prod_{i=1}^{I}\binom{N_i^1}{k_i^1}P(a_i)^{k_i^1}(1-P(a_i))^{N_i^1-k_i^1}\right)\left(\prod_{i=1}^{I}\binom{N_i^2}{k_i^2}P(a_i)^{k_i^2}(1-P(a_i))^{N_i^2-k_i^2}\right)$$

where the superscript ^{1,2} denotes data from cross-sections 1 and 2, respectively. The log-likelihood $\log(L)$ was maximised to obtain the maximum likelihood parameter estimate θ^* using the R statistical software. 95% confidence intervals were calculated using the likelihood ratio test. Profile likelihood plots are presented in Supplementary Figure S1.

Antibody acquisition model

The antibody acquisition model was fitted to data from both cross-sections by assuming the geometric mean antibody level at age a is $A(a)$ and that antibody levels are log-normally distributed in the cohort with standard deviation on the log scale σ.

For an individual k of age a_k with antibody level x_k, the likelihood that the model predicted geometric mean antibody level $A(a_k)$ fits the data is given by

$$L\left(\theta \middle| x_k, a_k\right) = \frac{1}{x_k \sigma \sqrt{2\pi}} e^{-\frac{\left(\log(x_k) - \log(A(a_k))\right)^2}{2\sigma^2}}$$

where θ is the parameter vector. For model 1 we have $\theta = \left(\alpha_0, r, \sigma\right)$, for model 2 $\theta = \left(\alpha_0, \alpha_c, t_c, r, \sigma\right)$, and for model 3 $\theta = \left(\alpha_0, \alpha_c, r, \sigma\right)$. The likelihood of the model fitting data from all individuals in both cross-sections is given by

$$L\left(\theta \middle| x^1_{1..K}, a^1_{1..K}, x^2_{1..K}, a^2_{1..K}\right) = \left(\prod_{k=1}^{K} \frac{1}{x^1_k \sigma \sqrt{2\pi}} e^{-\frac{\left(\log(x^1_k) - \log(A(a^1_k))\right)^2}{2\sigma^2}}\right)\left(\prod_{k=1}^{K} \frac{1}{x^2_k \sigma \sqrt{2\pi}} e^{-\frac{\left(\log(x^2_k) - \log(A(a^2_k))\right)^2}{2\sigma^2}}\right)$$

The log-likelihood $\log(L)$ was maximised to obtain the maximum likelihood parameter estimate θ^*. 95% confidence intervals were calculated using the likelihood ratio test. Profile likelihood plots are presented in Supplementary Figure S2.

Supplementary Figure S1 - Serocatalytic model profile likelihood plots

Profile likelihood plots for each of the estimated parameters showing the serocatalytic model uncertainty in the estimated parameter. Dashed lines indicate the 95% CI.

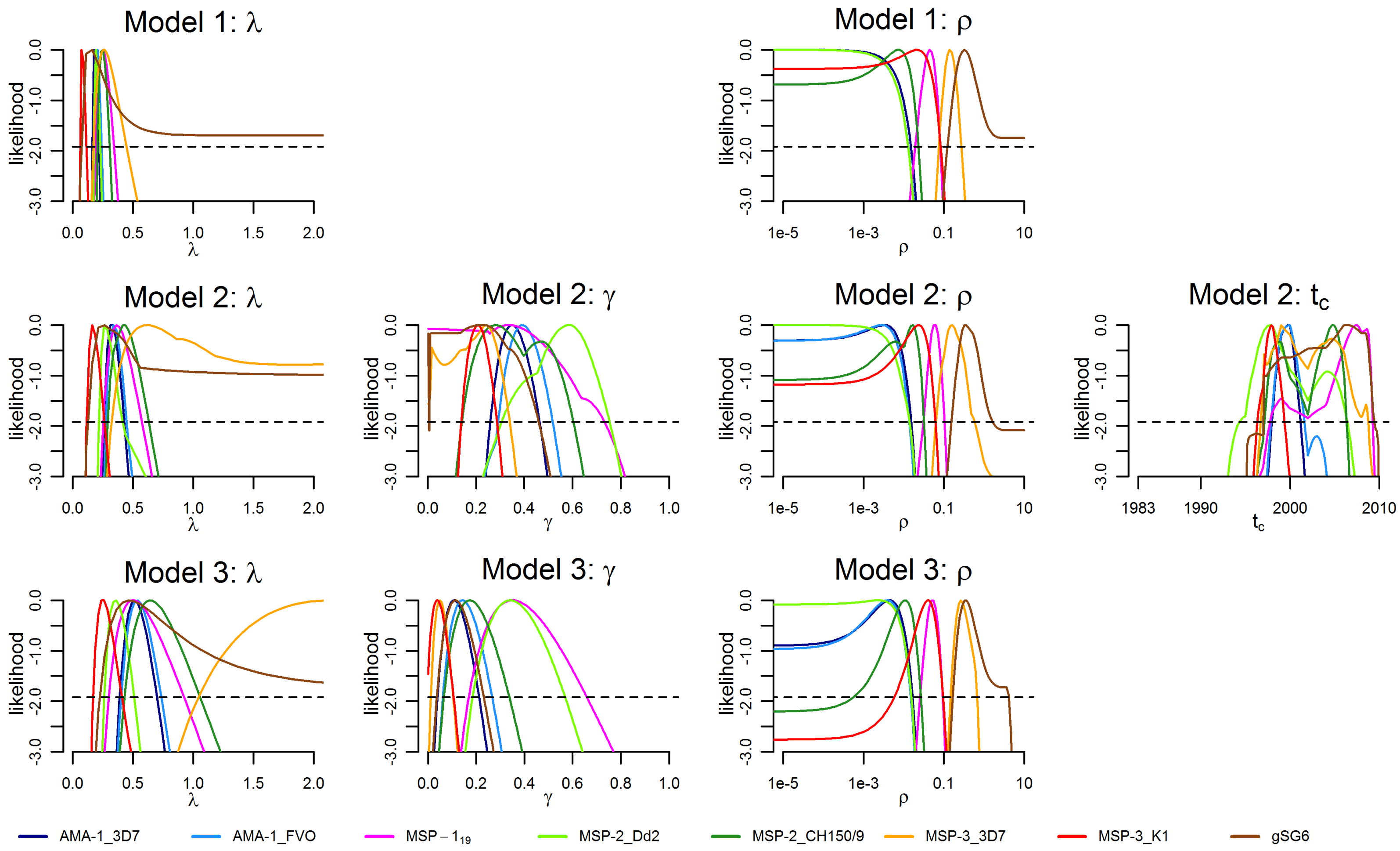

Supplementary Figure S2 - Antibody acquisition model profile likelihood plots

Profile likelihood plots for each of the estimated parameters showing the antibody acquisition model uncertainty in the estimated parameter. Dashed lines indicate the 95% CI.

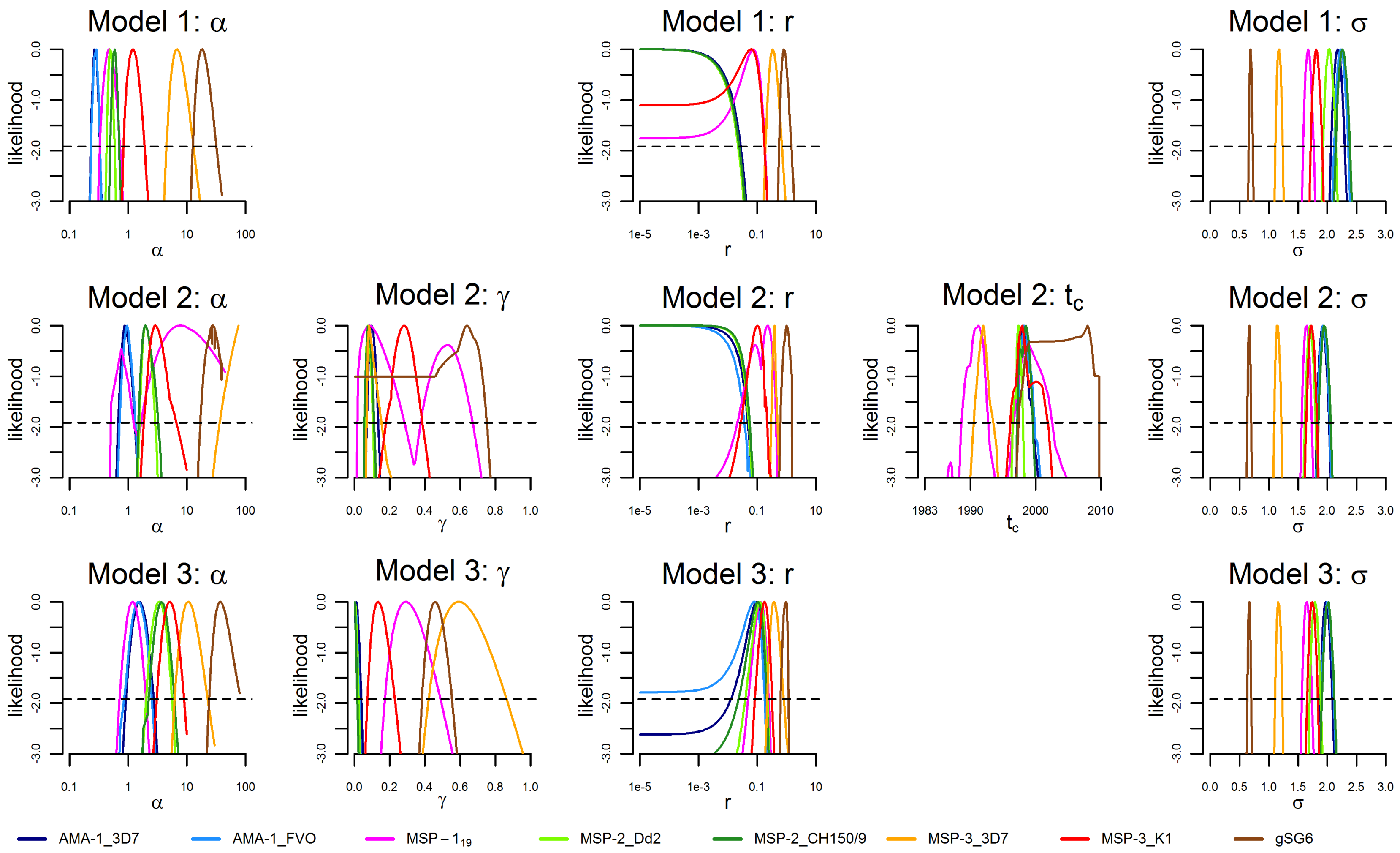

Supplementary Figure S3 - Distribution of antibody levels

Density distribution of log-transformed antibody levels in arbitrary units (AU). Red denotes measurements from the 1999 cross-section and green denotes measurements from the 2010 cross-section.

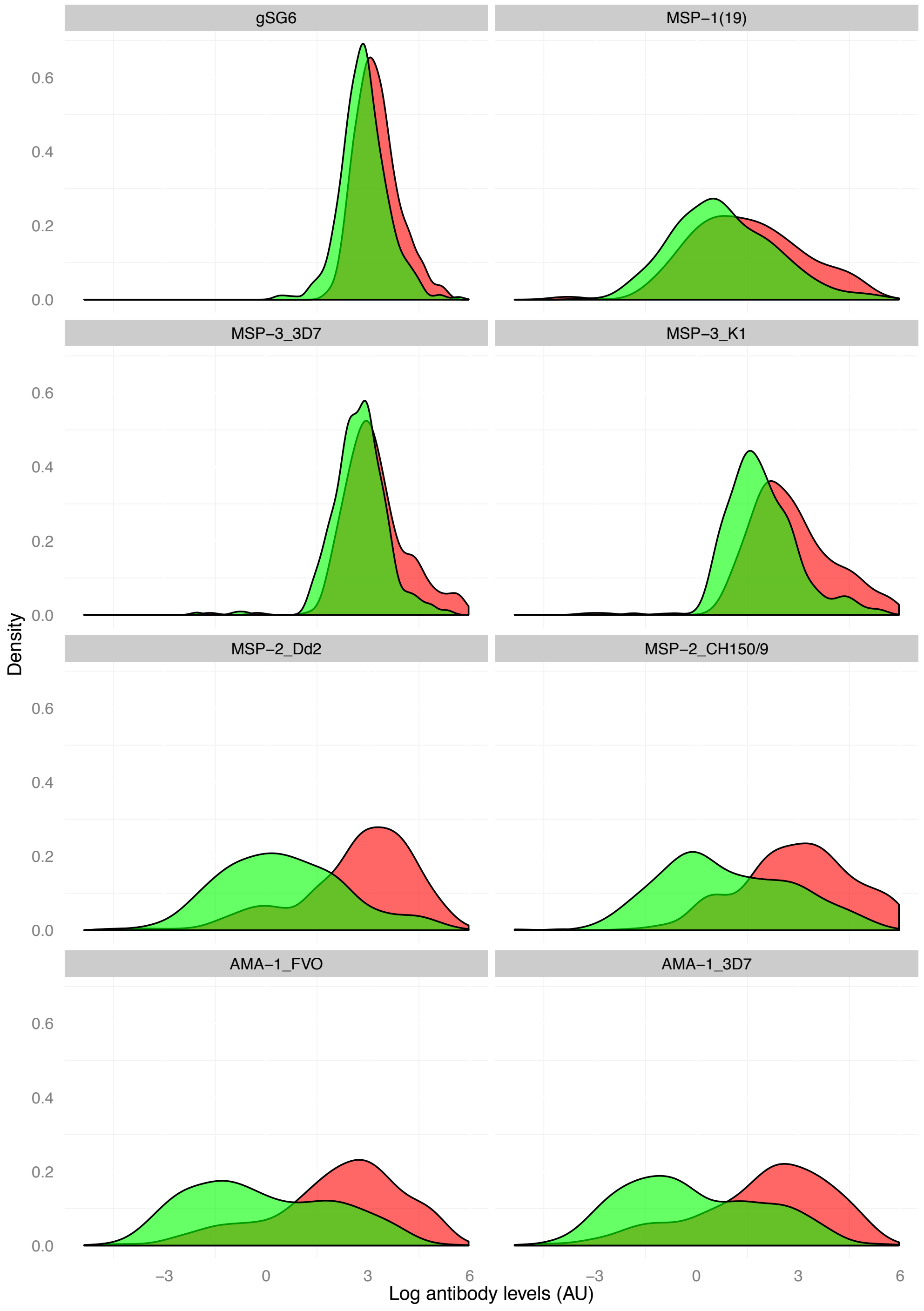

Supplementary Figure S4 - Correlation of antibody levels

Between antigen correlation of measured antibody levels (arbitrary units). Red points denote measurements from the 1999 cross-section and green points denote measurements from the 2010 cross-section.

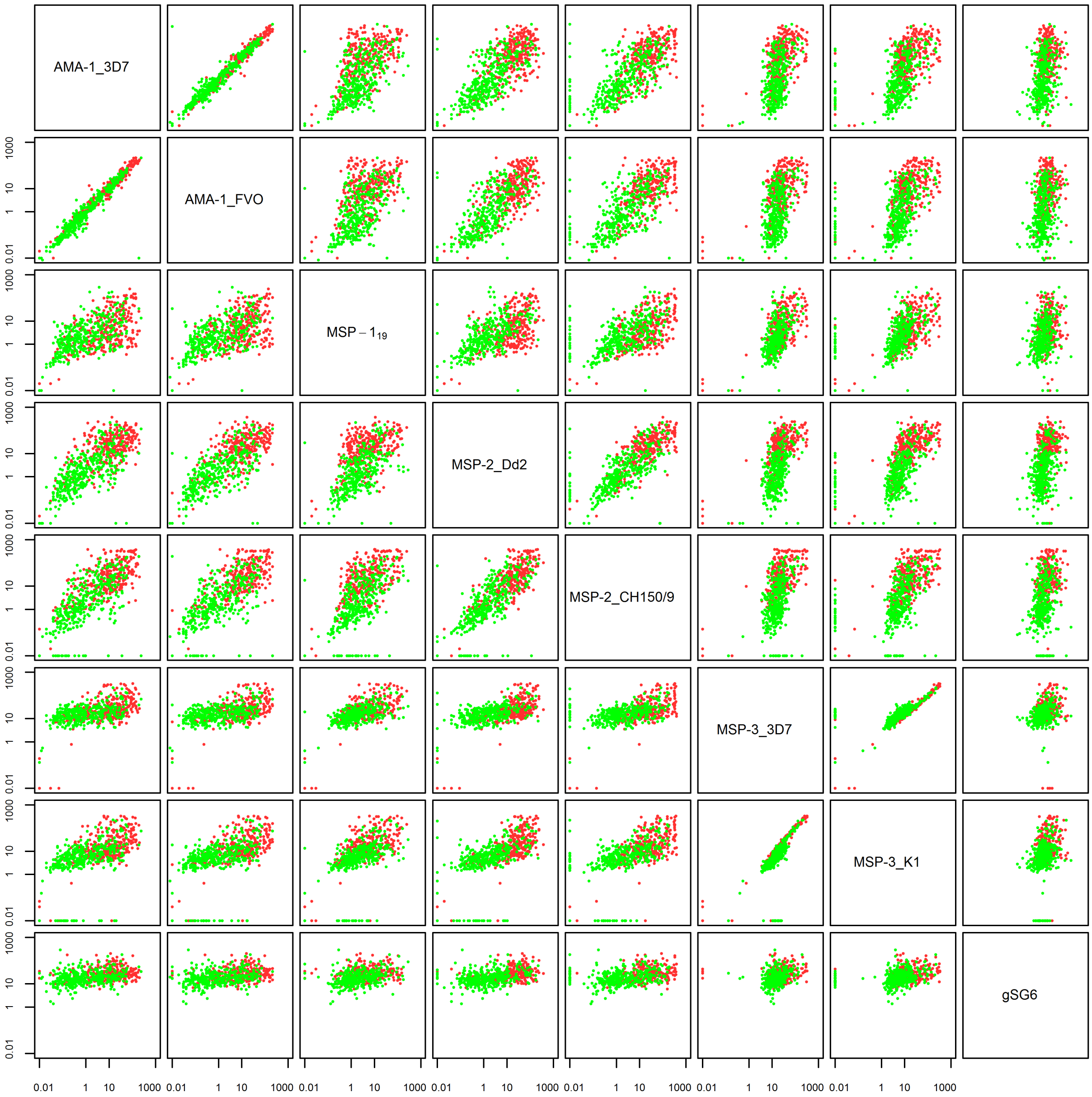

Supplementary Figure S5 - Best-fit serocatalytic models fitted to seroprevalence data defined using the alternative threshold for seropositivity.

The alternative threshold for seropositivity, generated using Gaussian mixture decomposition, was higher than the original threshold for all antigens and thus provided lower estimates of seroprevalence. The serocatalytic models fitted to these data estimated lower seroconversion rates, higher seroreversion rates and slightly larger reductions in transmission intensity. Parameter estimates were highly comparable with regards to the estimated time-point of change in transmission and, compared to the original threshold, the choice of an alternative threshold did not improve model performance. Model comparison using the AIC revealed that models 2 and 3 provided considerably better fits to data than model 1, however there was only sufficient information to determine that model 2 was the superior model for AMA-1 and MSP-2, whereas for the other antigens AIC values were similar between model 2 and 3. Black points denote the proportion of seropositive individuals and vertical bars denote 95% confidence intervals. Model 1: stable transmission (solid lines). Model 2: stepwise reduction in transmission (dotted lines). Model 3: linear reduction in transmission (dashed lines).

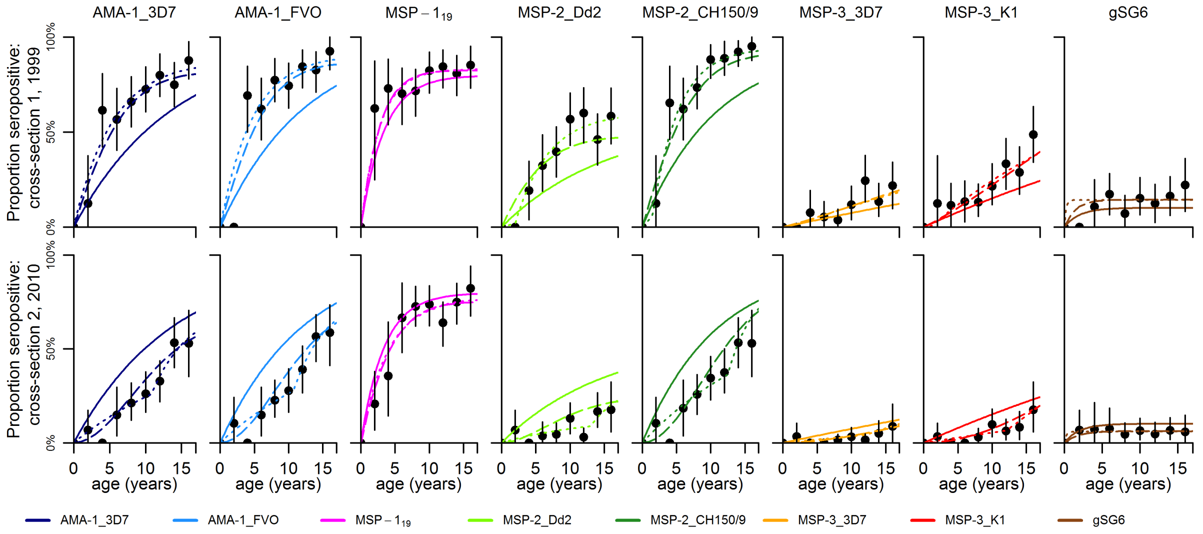

Supplementary Table S1 – Serocatalytic model parameter estimates (Seroprevalence defined using the alternative seropositivity threshold).

Antigen	Model	λ_0	γ	ρ	t_c	Log- likelihood	AIC
**AMA-1_3D7**	M1	0.07 (0.07, 0.10)	–	0.001 (0.0, 0.05)	–	-94.47	192.95
	M2	0.16 (0.14, 0.29)	0.19 (0.11, 0.28)	0.02 (0.0, 0.06)	1999 (1999, 2001)	-33.54	**75.08**
	M3	0.30 (0.21, 0.44)	0.0 (0.0, 0.04)	0.04 (0.01, 0.08)	–	-36.75	79.50
**AMA-1_FVO**	M1	0.08 (0.07, 0.12)	–	0.0 (0.0, 0.04)	–	-106.44	216.88
	M2	0.19 (0.14, 0.46)	0.18 (0.10, 0.25)	0.02 (0.0, 0.06)	1999 (1996, 2001)	-35.50	**79.01**
	M3	0.32 (0.24, 0.46)	0.0 (0.0, 0.04)	0.03 (0.004, 0.06)	–	-41.19	88.37
**MSP-1_{19}**	M1	0.24 (0.18, 0.34)	–	0.06 (0.03, 0.11)	–	-41.11	86.23
	M2	0.30 (0.21, 0.64)	0.65 (0.34, 0.85)	0.06 (0.03, 0.15)	1999 (1995, 2009)	-36.30	80.61
	M3	0.48 (0.29, 0.86)	0.36 (0.18, 0.67)	0.07 (0.04, 0.11)	–	-36.10	**78.20**
**MSP-2_Dd2**	M1	0.04 (0.03, 0.07)	–	0.03 (0.0, 0.12)	–	-96.00	196.0
	M2	0.14 (0.09, 0.41)	0.08 (0.04, 0.13)	0.07 (0.02, 0.15)	1997 (1995, 1998)	-32.3	**72.59**
	M3	0.19 (0.11, 0.56)	0.0 (0.0, 0.03)	0.12 (0.04, 0.33)		-43.87	93.74
**MSP-2_CH150/9**	M1	0.09 (0.11, 0.22)	–	0.004 (0.0, 0.04)	–	-116.54	237.08
	M2	0.25 (0.18, 0.53)	0.16 (0.10, 0.21)	0.011 (0.0, 0.03)	1997 (1997, 1998)	-33.89	**75.77**
	M3	0.32 (0.22, 0.53)	0.0 (0.0, 0.034)	0.016 (0.0, 0.046)	–	-41.53	89.06
**MSP-3_3D7**	M1	0.008 (0.0, 0.025)	–	0.0 (0.0, 0.12)	–	-35.88	75.77
	M2	0.01 (0.0, 5.0)	0.29 (0.05, 0.42)	0.0 (0.0, 0.14)	1998 (1987, 2003)	-25.14	58.28
	M3	0.02 (0.0, 0.06)	0.014 (0.0, 0.20)	0.024 (0.0, 0.21)	–	-26.03	**58.06**
**MSP-3_K1**	M1	0.016 (0.0, 0.04)	–	0.0 (0.0, 0.06)	–	-51.32	106.64
	M2	0.03 (0.0, 0.06)	0.18 (0.10, 0.32)	0.0 (0.0, 0.06)	1997 (1994, 2002)	-29.59	67.18
	M3	0.04 (0.03, 0.09)	0.0 (0.0, 0.10)	0.004 (0.0, 0.10)	–	-30.57	**67.14**
**gSG6**	M1	0.05 (0.01, 1.35)	–	0.45 (0.06, 1.0)	–	-32.87	69.73
	M2	0.69 (0.0, 5.0)	0.38 (0.0, 0.67)	1.3 (0.05, 4.16)	2007 (1991, 2009)	-26.84	61.68
	M3	0.20 (0.04, 1.39)	0.17 (0.04, 0.42)	0.64 (0.13, 3.0)	–	-26.72	**59.44**

Maximum likelihood parameter estimates and 95% confidence intervals for serocatalytic models fitted to cross-sectional age-specific seropositivity data using the alternative threshold for seropositivity. λ_0 is the seroconversion rate, γ $(=\lambda_c/\lambda_0)$ is the reduction in transmission, ρ is the seroreversion rate, t_c is the estimated time-point (calendar-year) of drop in transmission, *log-likelihood* is the maximised log-likelihood of the model and *AIC* is the Akaike Information Criterion value. A bold font indicates the smallest AIC for each of the antigens. Confidence Intervals were defined using profile-likelihood methods.

Supplementary Figure S6 - Sensitivity analysis using simulated data

Profile likelihood plots for parameter time of change (t_c) from serocatalytic and antibody acquisition model 2. The models have been fitted to simulated data for seroprevalence and levels of antibodies corresponding to two cross-sectional surveys and to four different sample sizes of 800, 400, 200 and 100 individuals, respectively. Data were simulated using the antibody acquisition model with a step change reduction in transmission to generate the geometric mean titre with variation between individuals on a population level described by a log-Normal distribution. Additional Gaussian noise was then added to the simulated data. The numbers refers to the number of samples and thus 200 would correspond to two cross-sections with 100 samples each. The exact change point was assumed to be 15 years prior to the sampling time-point (black point). The antibody acquisition model provided more accurate and precise estimates of the change point for all sample sizes.

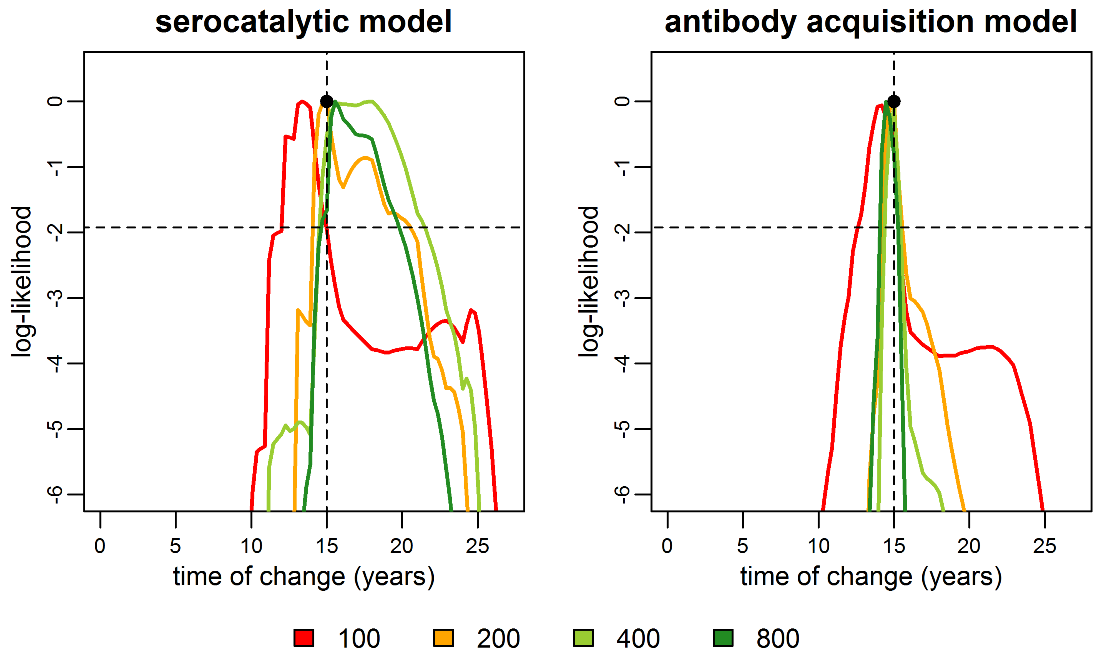

Supplementary Figure S7 - Sensitivity analysis using AMA-1_FVO data

Profile likelihood plots of parameter time of change (t_c) of serocatalytic and antibody acquisition model 2. The models have been fitted to the data for AMA-1_FVO. The sample size of n=668 corresponds to the full data set from the two cross-sections in 1999 and 2010. Sensitivity analysis was performed by randomly omitting samples from the full data set to obtain three reduced datasets with 400, 200 and 100 samples, respectively. The black point corresponds to the model estimates of time of change (t_c) from the full data set (n=668). The antibody acquisition model provided estimates with higher precision. Compared to the serocatalytic model both precision and accuracy of the antibody acquisition model were less affected by reducing the sample size.

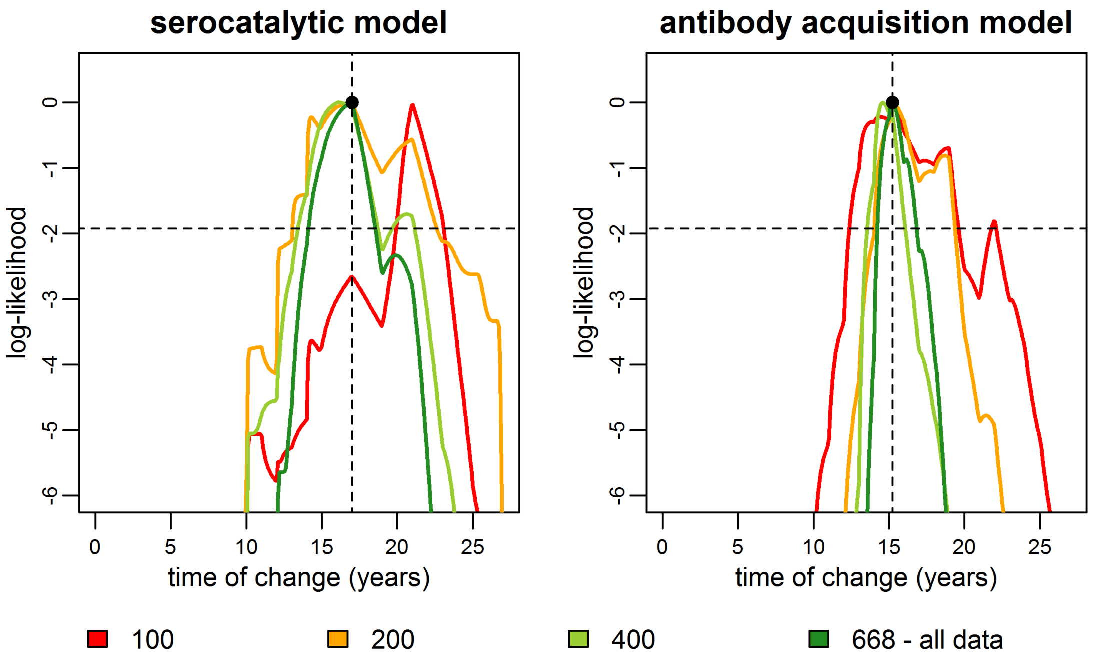

References

1. Rono, J. *et al.* Breadth of anti-merozoite antibody responses is associated with the genetic diversity of asymptomatic Plasmodium falciparum infections and protection against clinical malaria. *Clin. Infect. Dis.* **57,** 1409–1416 (2013).

2. Bolad, A. *et al.* Distinct interethnic differences in immunoglobulin G class/subclass and immunoglobulin M antibody responses to malaria antigens but not in immunoglobulin G responses to nonmalarial antigens in sympatric tribes living in West Africa. *Scand. J. Immunol.* **61,** 380–386 (2005).

3. Nagelkerke, N. J., Borgdorff, M. W. & Kim, S. J. Logistic discrimination of mixtures of M. tuberculosis and non-specific tuberculin reactions. *Stat. Med.* **20,** 1113–1124 (2001).

4. Cook, J. *et al.* Using serological measures to monitor changes in malaria transmission in Vanuatu. *Malar. J.* **9,** 169 (2010).

5. Everitt, B. S. & Hand, D. J. *Finite Mixture Distributions*. (Springer Netherlands, 1981). doi:10.1007/978-94-009-5897-5
